# *Acinetobacter baumannii* and *Klebsiella pneumoniae* Isolates Obtained from Intensive Care Unit Patients in 2024: General Characterization, Prophages, Depolymerases and Esterases of Phage Origin

**DOI:** 10.3390/v17050623

**Published:** 2025-04-26

**Authors:** Nadezhda V. Kolupaeva, Lyubov V. Kolupaeva, Peter V. Evseev, Yuriy P. Skryabin, Elena B. Lazareva, Tatyana V. Chernenkaya, Nikolay V. Volozhantsev, Anastasia V. Popova

**Affiliations:** 1 State Research Center for Applied Microbiology and Biotechnology, City District Serpukhov, Moscow Region, 142279 Obolensk, Russia; nadin.9830@mail.ru (N.V.K.); melstryder@yandex.ru (L.V.K.); sjurikp@gmail.com (Y.P.S.); nikvol@obolensk.org (N.V.V.); 2 Pirogov Russian National Research Medical University, 117997 Moscow, Russia; petevseev@gmail.com; 3 Sklifosovsky Research Institute for Emergency Medicine, 129090 Moscow, Russia; lazarevaeb@sklif.mos.ru (E.B.L.); chernenkayatv@sklif.mos.ru (T.V.C.)

**Keywords:** *Acinetobacter baumannii*, *Klebsiella pneumoniae*, genomes, prophage regions, tailspike depolymerase, tailspike esterase

## Abstract

*Acinetobacter baumannii* and *Klebsiella pneumoniae* are significant nosocomial pathogens worldwide. In this study, the general characterization of *A. baumannii* and *K. pneumoniae* isolates obtained from the blood of intensive care unit patients of the multidisciplinary scientific and practical center of emergency medicine from January to September 2024 was performed. Prophage regions and prophage-derived tailspike polysaccharide-depolymerizing or -modifying enzymes within these isolates were identified and characterized in detail using a refined workflow. The protocol, encompassing a comprehensive survey of all predicted bacterial proteins, revealed an average of 6.0 prophage regions per *Acinetobacter baumannii* genome, including regions putatively derived from filamentous phages, and 4.8 prophage regions per *Klebsiella pneumoniae* isolate. Analysis of these putative prophage regions indicated that most were related to previously isolated, yet unclassified, temperate phages infecting *A. baumannii* and *K. pneumoniae*. However, certain identified sequences likely originated from phages representing novel groups comparatively distant from known phages.

## 1. Introduction

*Acinetobacter baumannii* and *Klebsiella pneumoniae* are Gram-negative, catalase-positive, and oxidase-negative opportunistic bacteria that can cause a wide range of healthcare-associated infections [1]. These microorganisms are the members of the ESKAPE group (*Enterococcus faecium*, *Staphylococcus aureus*, *Klebsiella pneumoniae*, *Acinetobacter baumannii*, *Pseudomonas aeruginosa*, and *Enterobacter* spp.) which includes bacterial pathogens characterized by resistance to multiple classes of antibiotics and the ability to persist in hospital environments despite infection control procedures [2,3].

*A. baumannii* is a strictly aerobic, non-motile, non-fermentative coccobacillus belonging to the family *Moraxellaceae* [4,5]. *K. pneumoniae* is a facultative anaerobic, non-motile, rod-shaped bacterium belonging to the family *Enterobacteriaceae* [6]. Currently, these bacteria are regarded as significant nosocomial pathogens due to their tendency to develop multidrug-resistant (MDR), extensively drug-resistant (XDR), and pan-drug resistant (PDR) phenotypes [7,8]. The microorganisms are often associated with hospital-acquired pneumonia, wound and catheter-related urinary tract infections, peritonitis, meningitis, endocarditis, post-surgical complications, and bloodstream infections [1,9]. The spread of carbapenem-resistant *A. baumannii* and *K. pneumoniae* strains poses a serious threat to global public health, severely limiting the number of clinical treatment options. In this regard, the World Health Organization (WHO) assigned *K. pneumoniae* and *A. baumannii* to the group of critical priority microorganisms for the development of new antibacterial agents [10].

Most of clinically relevant *A. baumannii* and *K. pneumoniae* strains produce capsular polysaccharides (CPSs), which are one of the most important virulence factors of these microorganisms and form a thick protective layer around the bacterial cells, allowing them to avoid the actions of the host immune system [8,11,12]. To date, based on the analysis of the *A. baumannii* and *K. pneumoniae* genomic sequences deposited in the NCBI database, more than 240 [13] and 134 [14] variants of capsule biosynthesis gene loci (KL) have been bioinformatically predicted, respectively.

The genomes of many lytic bacteriophages (phages) infecting *A. baumannii* and *K. pneumoniae* contain genes encoding structural proteins with polysaccharide-depolymerizing or modifying activities [15,16]. These proteins are usually highly specific tailspike enzymes that are responsible for degrading or modifying CPSs with a certain structure during the attachment and adsorption of a phage to a bacterial host cell [17]. The genetic material of prophages integrated into bacterial genomes can also contain genes encoding tailspike depolymerases or esterases [18].

The aim of this work was to identify, bioinformatically characterize, and categorize prophage regions and different prophage-derived tail enzymes in the genomes of nosocomial *A. baumannii* and *K. pneumoniae* isolates obtained from patients of the multidisciplinary scientific and practical center of emergency medicine (Moscow, Russia) during 2024. To achieve this, we proposed a workflow protocol designed to identify prophage regions and their tail spike depolymerases and esterases within the genomic data of the studied isolates. This algorithm, successfully applied to our local isolate collection, offers a scalable approach for analyzing additional genomic data to identify prophages and their potential for encoding CPS-degrading/modifying enzymes.

## 2. Materials and Methods

### 2.1. Bacterial Isolates and Culturing

*A. baumannii* (n = 15) and *K. pneumoniae* (n = 52) isolates were obtained from the blood of patients of the intensive care unit of the multidisciplinary scientific and practical center of emergency medicine (Moscow, Russia) from January to September 2024. *A. baumannii* and *K. pneumoniae* isolates from the same patient were collected at least one week apart. The isolates collected on the same date were obtained from different intensive care unit patients. Species identification was initially performed using a MALDI-TOF Biotyper system (Bruker Daltonics, Bremen, Germany). Bacterial cells were grown at 37 °C on Luria–Bertani agar (Difco Laboratories, Detroit, MI, USA) and Nutrient Medium No. 1 (SRCAMB, Obolensk, Moscow region, Russia). Bacterial isolates were stored in 20% glycerol at −80 °C. All isolates were deposited to the State Collection of Pathogenic Microorganisms and Cell Cultures (SCPM-Obolensk) under the corresponding accession numbers.

### 2.2. Whole-Genome Sequencing and Assembly

The procedures of DNA isolation, whole-genome sequencing (WGS), and assembly were performed and described in our previous work [19]. Briefly, WGS was performed using the DNBSEQ-G400 (MGISEQ-2000) platform (BGI, Shenzhen, China) with prefragmentation of DNA molecules using the BioRuptor system (Diagenode, Denvile, NJ, USA), the MGIEasy Universal DNA Library Prep Set (Wuhan MGI Tech Co., Ltd., Wuhan, China), and the MGISEQ-2000 PE150 High-throughput Sequencing Kit (Wuhan MGI Tech Co., Ltd., Wuhan, China). The assemblies of the genomes were obtained using Unicycler v. 0.5.0 software (The University of Melbourne, Melbourne Australia) [20] with default settings that included primary filtering and quality control. In this work, the completeness and metrics of *A. baumannii* and *K. pneumoniae* genome assemblies were assessed using CheckM2 v. 1.1.0 [21] (Table S1).

### 2.3. Identification of Capsule Synthesis Loci and Multilocus Sequence Types

The identification of capsule biosynthesis loci (K loci, KL) in the genome data was performed using Kaptive (https://kaptive-web.erc.monash.edu/, accessed on 20 January 2025) [13,22]. Multilocus sequence typing (MLST) was carried out by submitting genome assemblies to the PubMLST database available at https://pubmlst.org/organisms/, accessed on 22 January 2025.

### 2.4. Antimicrobial Susceptibility

Susceptibility to antimicrobials (AMs) of 11 functional groups—penicillins, penicillin/beta-lactam inhibitors, carbapenems, monobactams, cephalosporins, fluoroquinolones, aminoglycosides, sulfonamides, fosfomycins, trimethoprims, and polymyxins—were determined using Vitek-2 Compact instrument using AST *n*-360 card (BioMerieux, Paris, France) (Table S2). The results were interpreted according to the European Committee on Antimicrobial Susceptibility Testing, Breakpoint tables for interpretation of MICs and zone diameters. V.15.0. 2025 (http://www.eucast.org, access date: 3 March 2025). The isolates were categorized as multidrug-resistant (MDR), non-susceptible to ≥1 agent in ≥3 antimicrobial groups; extensively drug-resistant (XDR), non-susceptible to ≥1 agent in all but ≤2 groups; and pandrug-resistant (PDR), non-susceptibility to all agents, according to the criteria proposed by Magiorakos et al. [23].

### 2.5. Search and Annotation of Prophages and Search for Phage Tail Depolymerases and Esterases

Prophage regions were identified using PHASTEST [24] with default settings. Bacterial genomes were annotated using Bakta [25] with default settings. Additionally, phage sequences were annotated separately by predicting and validating open reading frames (ORFs) using Glimmer v3.02b [26], Prodigal v2.6.1 [27], and manual curation to ensure accuracy. Functions were assigned to ORFs using a BLAST search against a custom phage protein database compiled from annotated phage GenBank sequences, an InterProScan search [28] via Geneious Prime v2025.0.3 (Biomatters, Inc., Auckland, New Zealand), an HHblits search with HHsuite v3.3.0 [29], and the HHpred server (https://toolkit.tuebingen.mpg.de, accessed 1 December 2024) [30]. The presence of tRNA-coding regions in prophage sequences was checked using tRNAscan-SE [31]. The resulting genome map was visualized using clinker [32]. Individual phage proteins were identified using translated sequences of putative genes and HHblits v3.3.0 [29] with default settings and the databases pdb70_from_mmcif_2023-06-18, pfama-v35, and uniprot_sprot_vir70_Nov_2021, followed by a keyword search for “capsid protein”, “terminase”, “tail fiber”, “tailspike”, and “tail spike” using a custom script, and then verified by manual inspection.

### 2.6. Bioinformatic Analysis

Multiple sequence alignments of nucleotide and amino acid sequences were generated using MAFFT version 7.48 [33] with the default settings and the L-INS-i algorithm. The concatenated alignments of characteristic bacterial proteins were obtained using GTDB-Tk v2.4.0 [34]. Phylogenetic analysis of the aligned sequences was performed using IQ-TREE v2.2.5 [35] with the command-line parameters “-m TEST -ninit 1000 -bb 1000”, which included a bootstrap analysis with 1000 replicates to evaluate the robustness of the phylogenetic tree. These parameters employed ModelFinder [36] to determine the optimal substitution model. Intergenomic comparisons of phages were conducted using VIRIDIC v1.1 [37] (https://rhea.icbm.uni-oldenburg.de/viridic, accessed 10 December 2024) with default settings. Bacterial average nucleotide identity (ANI) was calculated using FastANI [38]. The ANI heatmap was constructed using ANIclustermap (https://github.com/moshi4/ANIclustermap, accessed 1 December 2024). A proteomic tree was constructed using the ViPTree server [39] (https://www.genome.jp/viptree/, accessed 10 December 2024) with default settings. All phylogenetic trees were visualized using iTOL v7 [40]. Protein structures were predicted using AlphaFold 3 (AF3) [17] and visualized with PyMOL v2.5.4 (Schrödinger Inc., New York, NY, USA). The highest-ranked AF3 models were used for structural analyses and comparisons, and structural similarity was assessed using the DALI Z-score [41]. The presence of genes encoding antibiotic resistance was determined through bioinformatic analysis using the Comprehensive Antibiotic Resistance Database (CARD) v3.2.7 [42].

## 3. Results

### 3.1. General Characterization of A. baumannii and K. pneumoniae Isolates

*A. baumannii* and *K. pneumoniae* isolates were obtained from the blood of patients of the intensive care unit of the multidisciplinary scientific and practical center of emergency medicine from January to September 2024. The WGS of these strains was performed and described previously [19]. By WGS and MLST analyses *A. baumannii* isolates were assigned to three sequence types (STs): ST2 (n = 6), ST78 (n = 6), and ST19 (n = 3) in the *A. baumannii* Institute Pasteur MLST scheme (Table 1). It was found that *A. baumannii* genomes carry four different KLs: KL3 (n = 6), KL235 (n = 5), KL17 (n = 3), and KL49 (n = 1). Among *K. pneumoniae* isolates, nine STs: ST395 (n = 16), ST39 (n = 14), ST147 (n = 7), ST512 (n = 6), ST101 (n = 4), ST23 (n = 2), ST15 (n = 1), ST218 (n = 1), and ST307 (n = 1), and twelve KLs: KL23 (n = 11), KL48 (n = 10), KL64 (n = 6), KL107 (n = 6), KL2 (n = 5), KL17 (n = 4), KL20 (n = 3), KL1 (n = 2), KL39 (n = 2), KL57 (n = 1), KL102 (n = 1), and KL112 (n = 1), were identified.

### 3.2. Phylogenomic Characterization of Isolates

#### 3.2.1. Phylogenomic Characterization of *A. baumannii* Isolates

**Figure 1:**
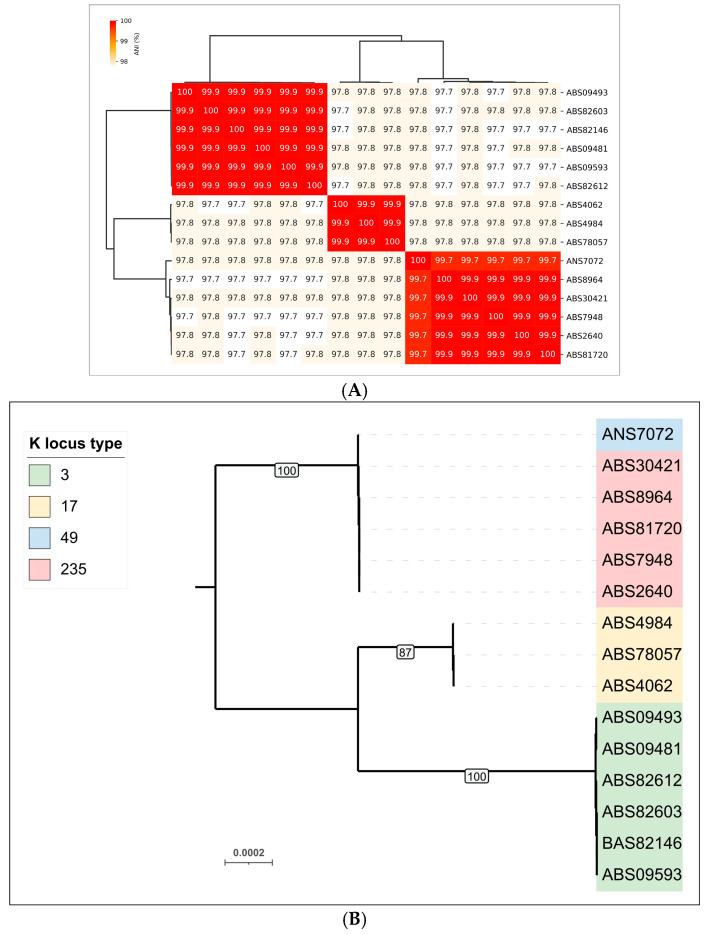
(**A**) Heatmap and dendrogram depicting the relationships among *A. baumannii* isolates based on average nucleotide identity (ANI). The color scale represents the percentage identity between whole-genome sequences, ranging from high (red) to low (white). (**B**) Maximum-likelihood phylogenetic tree based on concatenated amino acid sequences of conserved proteins, constructed using GTDB-Tk. Bootstrap values are shown near the nodes. The scale bar indicates 0.0002 estimated substitutions per site, and the tree is midpoint-rooted. *A. baumannii* KL type is indicated in the legend.

Average nucleotide identity (ANI) calculations and clustering of the genomic sequences of 15 *A. baumannii* isolates indicated that they can be grouped into three clusters (Figure 1A). This clustering is consistent with the results of phylogenetic analysis based on concatenated alignments of conserved proteins, as performed by the GTDB-Tk pipeline for objective taxonomic classification of bacterial and archaeal genomes (Figure 1B). Predictions of KL type generally agreed with phylogenomic clustering; only in one case did a cluster consisting mainly of isolates carrying KL235 also contain a KL49-encoded isolate (ANS7072).

#### 3.2.2. Phylogenomic Characterization of *K. pneumoniae* Isolates

**Figure 2:**
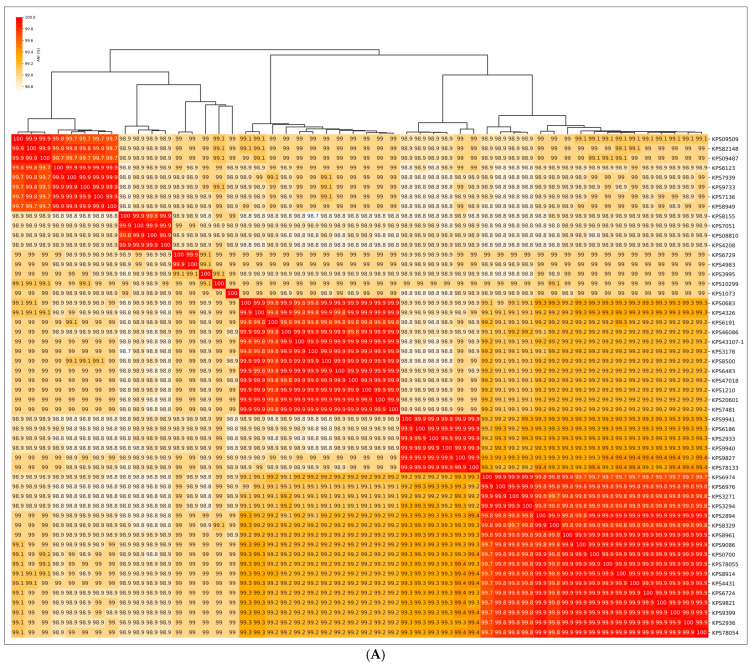

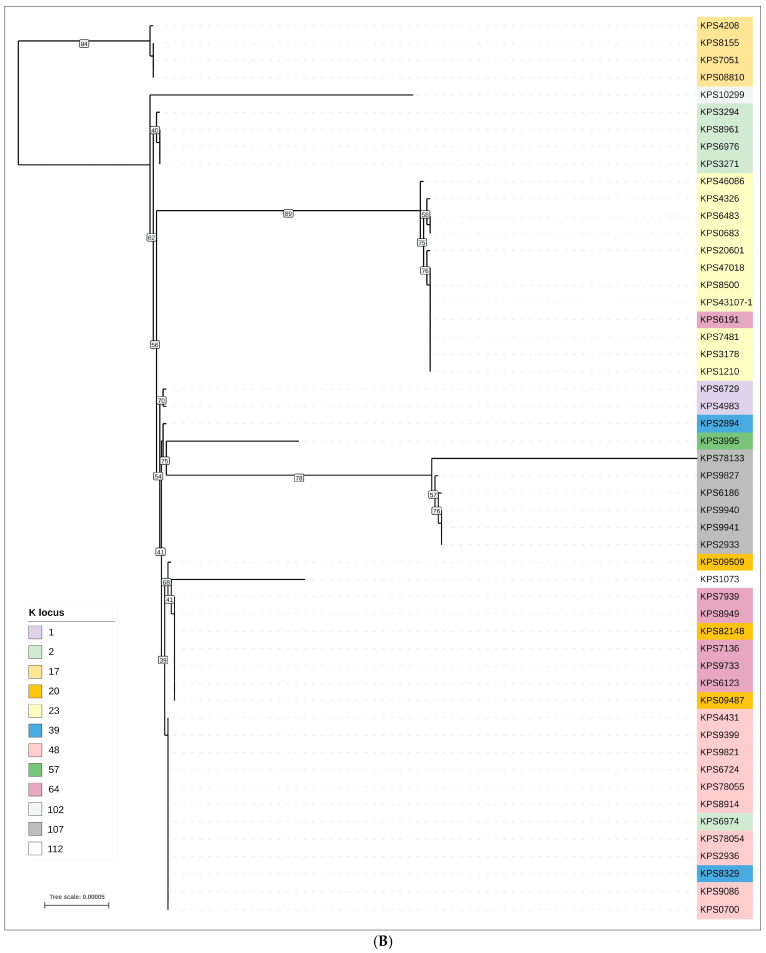
(**A**) Heatmap and dendrogram depicting the relationships among *K. pneumoniae* isolates based on average nucleotide identity (ANI). The color scale represents the percentage identity between whole-genome sequences, ranging from high (red) to low (white). (**B**) Maximum-likelihood phylogenetic tree based on concatenated amino acid sequences of conserved proteins, constructed using GTDB-Tk. Bootstrap values are shown near the nodes. The scale bar indicates 0.00005 estimated substitutions per site, and the tree is midpoint-rooted. *K. pneumoniae* KL type is indicated in the legend.

ANI comparisons and GTDB-Tk analysis of 52 *K. pneumoniae* isolates indicated less genomic variability compared to *A. baumannii* (Figure 2). These analyses grouped the *K. pneumoniae* isolates into nine clusters, including three singleton clusters. As observed for *A. baumannii*, there was a distinct, but not absolute, correlation between KL type and position in the GTDB-Tk tree or ANI-based clustered heatmap. The *K. pneumoniae* isolates exhibited greater KL diversity at lower degrees of genomic similarity than the *A. baumannii* isolates.

### 3.3. Identification of Prophage Regions and Phage Tail Depolymerases and Esterases in Bacterial Genomes

#### 3.3.1. Protocol

**Figure 3:**
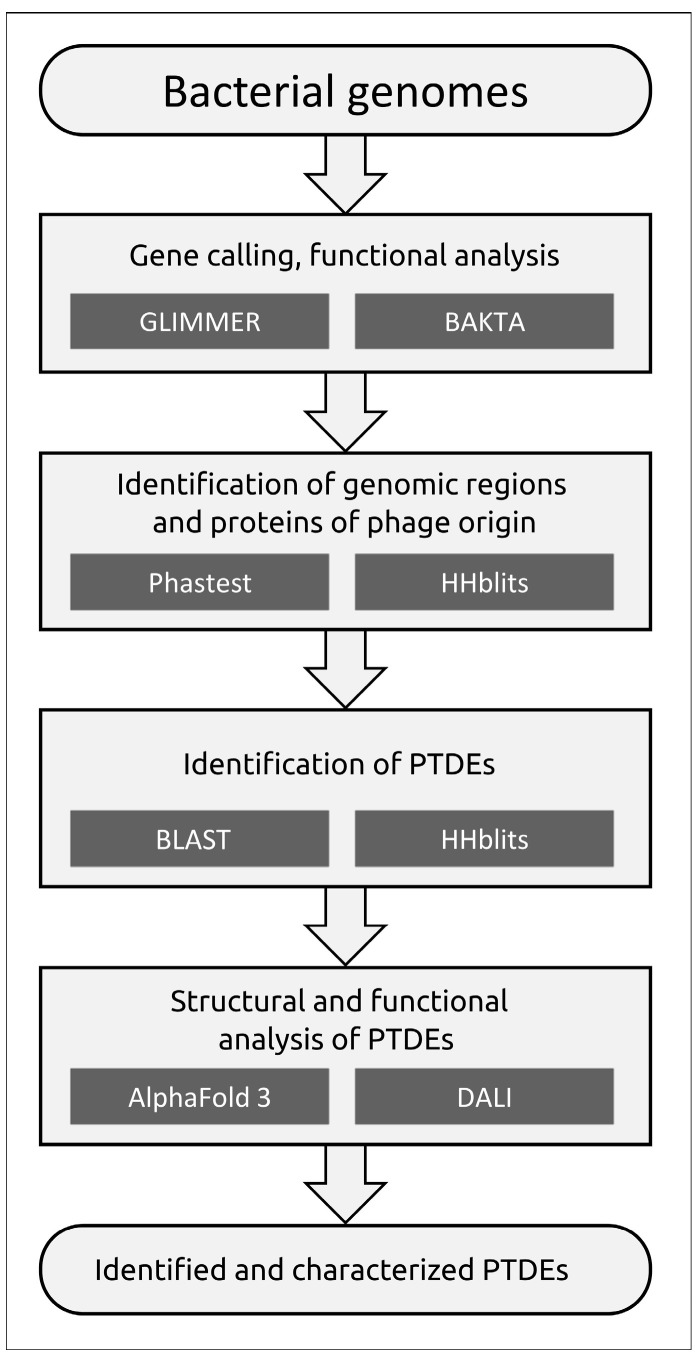
Workflow for identification and structural analysis of phage tailspike depolymerases and esterases (PTDEs).

Prophage regions were initially identified using PHASTEST [24], a well-established prophage search tool, and a sensitive Hidden Markov Model (HMM)-based search (HHblits [29]) to detect genomic regions containing genes encoding the phage major capsid protein (MCP) and the terminase large subunit (TLS). Comparison of the results revealed that PHASTEST failed to identify some genes encoding HK97-fold MCPs and TLSs characteristic of tailed phages (class *Caudoviricetes*) and MCPs of filamentous bacteriophages (class *Faserviricetes*). Therefore, prophage regions were ultimately identified through manual inspection of regions flagged by both PHASTEST and HHblits. Prophage-derived enzymes with polysaccharide-depolymerizing or modifying activities were identified via BLAST and HHblits searches (Figure 3). The subsequent analysis was limited to depolymerases and esterases (PTDEs, phage tailspike depolymerases and esterases) found within prophage receptor-binding proteins (RBPs).

#### 3.3.2. General Characterization of Prophage Regions in *A. baumannii* Genomes

**Figure 4:**
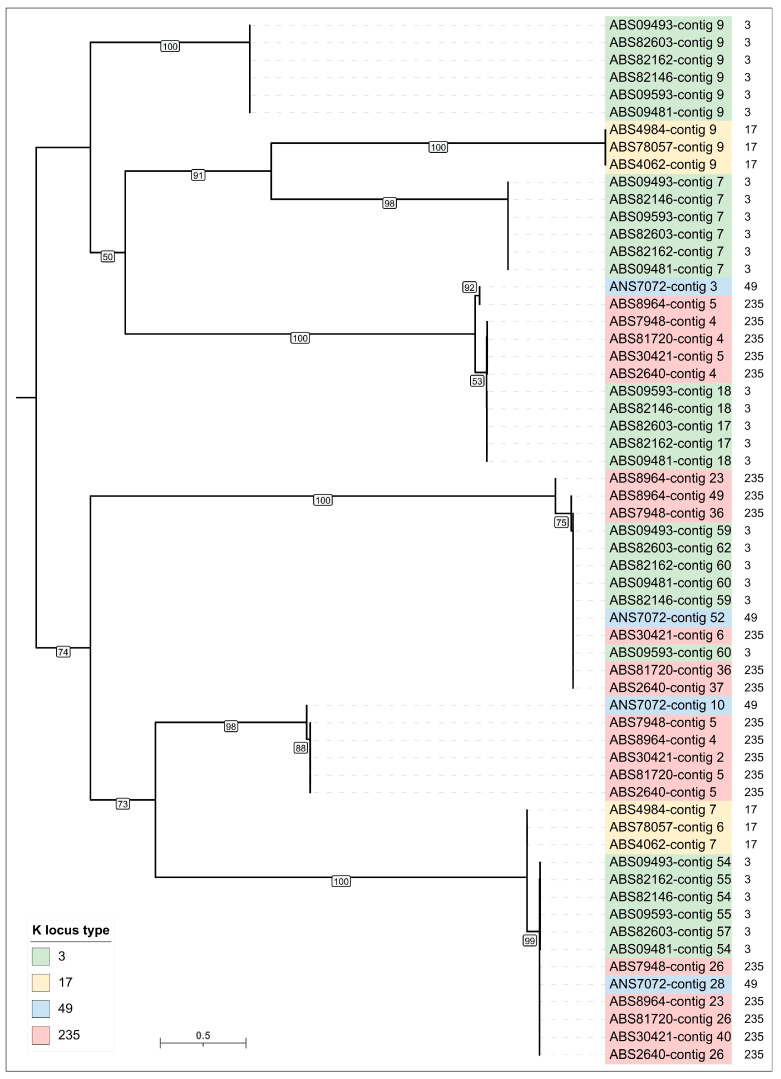
Maximum-likelihood phylogenetic tree based on amino acid sequences of MCP encoded in *A. baumannii* prophage regions. Bootstrap values are shown near the nodes. The scale bar indicates 0.5 estimated substitutions per site, and the tree is midpoint-rooted. *A. baumannii* KL type is indicated in the legend. Prophage regions are named according to the contig of the genome assembly in which they were identified.

**Figure 5:**
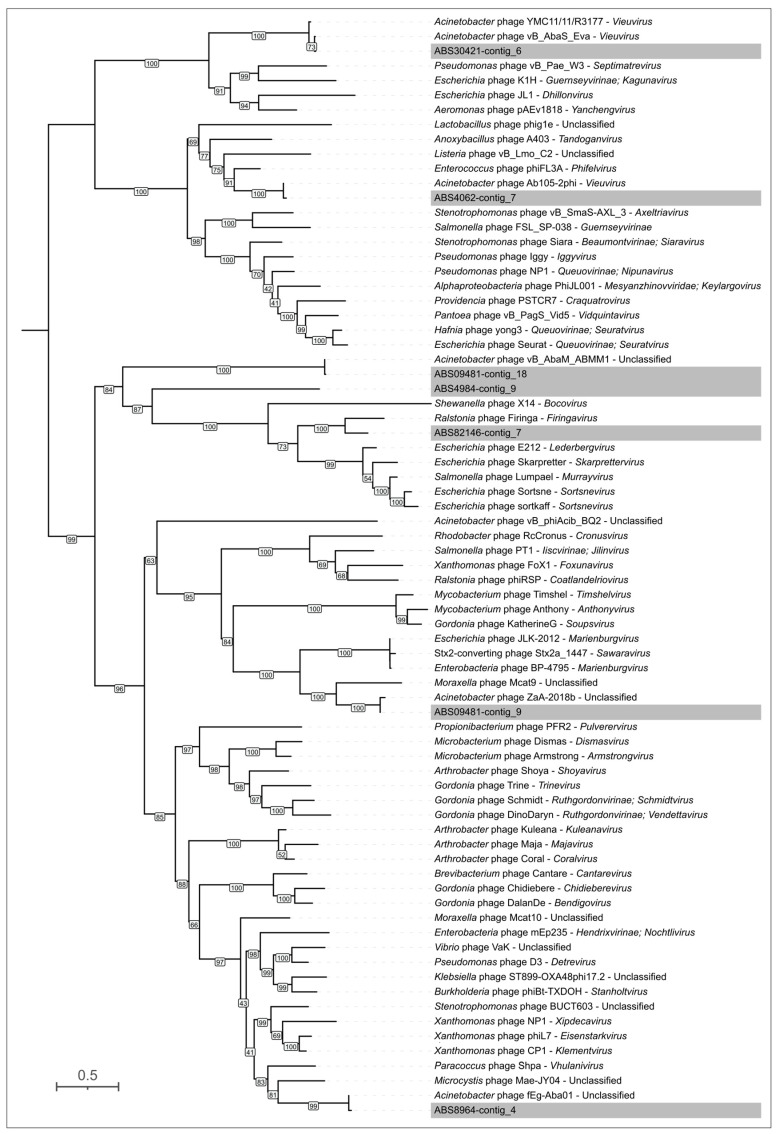
Maximum-likelihood phylogenetic tree based on amino acid sequences of MCP encoded in *A. baumannii* prophage regions and related phages. NCBI taxonomy is shown to the right of phage names. Bootstrap values are shown near the nodes. The scale bar indicates 0.5 estimated substitutions per site, and the tree is midpoint-rooted. Representative prophage regions are colored gray.

A combined search across the genomes of 15 *A. baumannii* isolates involving the identification of prophage regions using PHASTEST and the detection of MCP and TLS using HHpred revealed 90 genomic regions containing prophage-derived proteins. Of these, PHASTEST identified 48 prophage regions, while additional searches identified 42. Prophage regions were often similar or nearly identical in closely related isolates. The number of identified prophage regions was also consistent across closely related isolates, except for ABS8964. Specifically, seven prophage regions were found in genomes belonging to clusters that combine *A. baumannii* isolates carrying KL 3 and KL49 (Figure 2). Six prophage regions were identified in isolates of KL type 17, and four prophage regions were detected in all genomes of isolates assigned to KL type 235, except of ABS8964. Importantly, closely related putative prophages were found in *A. baumannii* isolates of different capsular types. Out of the 90 regions, 60 contained genes (or their remnants) that code for MCPs and TLSs, enabling clustering and preliminary classification of the prophages using sequences of these conserved proteins and sequence of homologs of these proteins found using BLAST searches (Figure 4 and Figure 5, Supplementary Figures S1 and S2). Notably, the topologies of MCP and TLS are incongruent, indicating a complex evolutionary history of viral proteins accompanied by events of genetic exchanges [43].

Despite the identification of a relatively large number of prophages, phylogenetic analysis revealed only a few distinct prophage groups. Some of these groups were ubiquitous across nearly all *A. baumannii* genomes, whereas others were specific to closely related bacterial isolates (Figure 4 and Figure 5). The majority of the identified prophages were closely related to previously isolated phages known to infect *A. baumannii*, including those classified within the *Vieuvirus* genus and unclassified *Acinetobacter* phages (e.g., 5W [44], vB_AbaS_Eva [45], vB_AbaM_ABMM1 [46], ZaA-2018b, fEg-Aba01 [47], vB_AbaS_TRS1 [48]). However, six identical prophages found in the genomes of all *A. baumannii* isolates carrying KL3 exhibited greater similarity to the *Ralstonia* phage Firinga [49] of the *Firingavirus* genus (based on similarity of their MCPs) and the *Sodalis* phage phiSG1 (based on similarity of their TLSs) rather than to *Acinetobacter* phages.

Interestingly, an HHblits search detected sequences of filamentous phage origin in isolates ABS4984, ABS4062, and ABS78057, although these sequences appeared to be incomplete (3998 nucleotides). A BLAST search using predicted structural protein sequences did not identify distinct homologs other than those in *A. baumannii* and *Alkanindiges* sp. However, homologs of the zonular occludens toxin (ZOT) family protein, which are characteristic of inovirus-like phages, were found among the isolated phages assigned to the *Inovirus* genus (family *Inoviridae*, order *Tubulavirales*). This may indicate a higher conservation of the ZOT-like proteins, making them suitable as signature proteins for phylogenetic studies.

**Figure 6:**
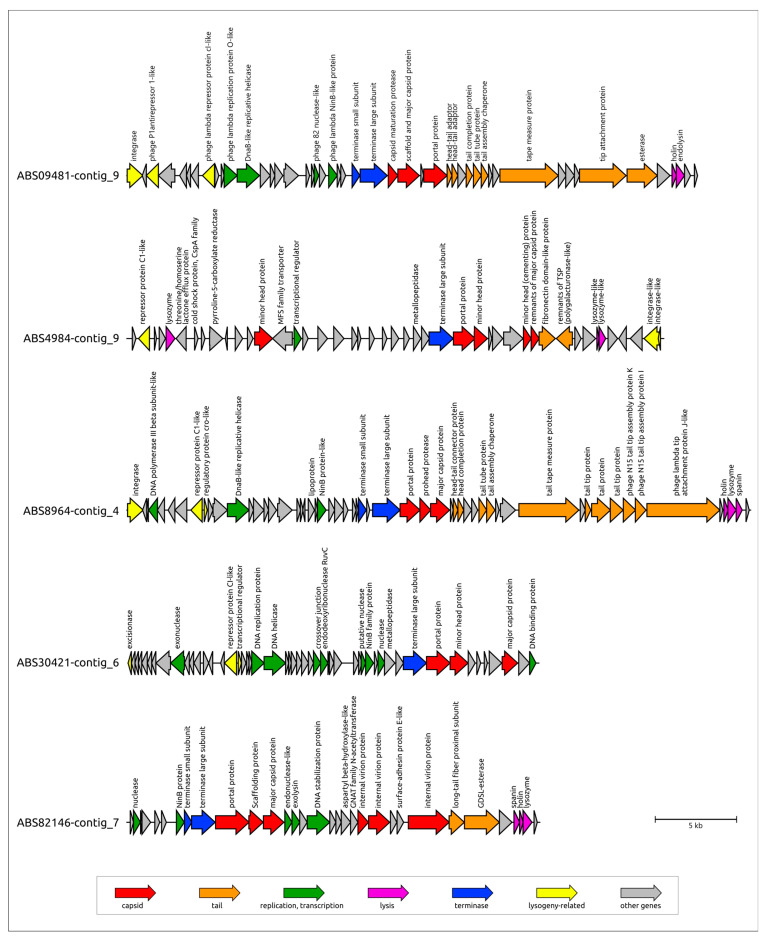

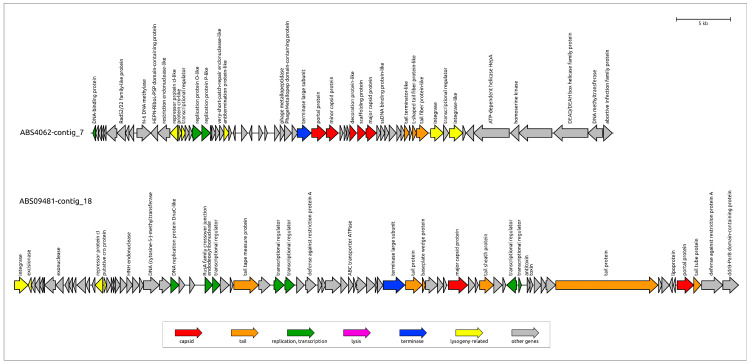
Genetic maps of prophage regions revealed in the genomes of different *A. baumannii* isolates. Gene annotations and predicted functions are indicated by labels and a legend. Arrows show the direction of transcription for each gene. The scale bar represents the length of the nucleotide sequence.

Seven prophage regions representing different groups in the phylogenetic trees were annotated using BLAST and HMM-based methods (Table 2, Figure 6, Supplementary File S1). This is a region found in contig_9 of the ABS09481 genome assembly (contigs were numbered in descending order of size) and labeled as ABS09481-contig_9, and these are regions designated as ABS8964-contig_4, ABS09481-contig_18, ABS4984-contig_9, ABS30421-contig_6, ABS82146-contig_7, and ABS4062-contig_7 ABS09481-contig_9 (Figure 6). Notably, the putative prophage regions ABS4984-contig_9, ABS8964-contig_4, and ABS09481-contig_18 were flanked by integrase genes. Furthermore, a tRNA(Gly) gene was present upstream of the integrase gene in the prophage region ABS4984-contig_9, as well as in all other prophage regions belonging to its cluster.

ABS09481-contig_9 confidently corresponds to a complete temperate phage genome, as determined by comparing its nucleotide sequence with the genomic sequences of previously isolated phages deposited in GenBank. Prophage ABS09481-contig_9 appears to be a siphovirus with a rather short genome of ~35 kb, related to several unclassified *Acinetobacter* phages and several temperate siphoviruses infecting *Moraxella catarrhalis* [50]. In the tail module, this prophage contains a gene that encodes a product sharing similarity with O-acetylesterases (HHpred probability 100%), suggesting that the corresponding protein may participate in the adsorption stage of infection.

ABS8964-contig_4 also appears to represent the complete genome of a temperate phage. Comparison of the tail modules of ABS09481-contig_9 and ABS8964-contig_4 revealed differences in the adsorption apparatuses of the corresponding phages: ABS8964-contig_4 lacks a gene of an esterase domain-containing protein but encodes another protein reminiscent of the tail fiber and tailspike proteins (TFPs and TSPs) found in other phages, indicating distinct mechanisms of adsorption.

Another prophage region found in ABS09481 (specifically in contig_18) contains all genomic modules present in a typical temperate phage and is closely related to the unclassified *Acinetobacter* phage vB_AbaM_ABMM1. A BLAST search did not reveal homologs of its major capsid protein (MCP) in phages other than vB_AbaM_ABMM1. However, similarities were found between the terminase large subunits (TLSs) of this prophage (and vB_AbaM_ABMM1) and the terminase B (PacB) proteins of P1-like phages. Prophage ABS09481-contig_18 appears to represent a myovirus, because it carries a gene encoding a tail sheath protein. An intriguing feature of this prophage is the potential presence of an advanced defense system, including antirestriction enzymes and a toxin-antitoxin system. This prophage region may represent an intact prophage.

In contrast, the prophage region found in isolate ABS4984 (contig_9, Figure 6) likely represent a domesticated prophage. This prophage region is characterized by an abundance of apparently degraded genes, which are shortened compared to their original versions. For instance, a close relative of the phage that formed the prophage region in the genome of isolate ABS4984 (contig_9), *Acinetobacter* phage 5W, has a very similar TLS (86% amino acid pairwise identity). However, the MCP gene in the ABS4984_contig_9 prophage has degraded to a remnant of 163 amino acids (aa). Additionally, the original lysozyme gene appears to have been split into two fragments, presumably encoding proteins of 45 and 136 aa, corresponding to the N-terminal and C-terminal parts of a full-sized 181 aa lysozyme, both of which show homology to the prophage fragments. A similar degradation was observed in the integrase gene, which was also split into two fragments. The degradation of genes in this prophage region is further evident from several long intergenic regions of ~500 bp, which are absent in phage 5W and related phages.

The completeness of the remaining representative prophages (labeled as ABS30421-contig_6, ABS82146-contig_7, and ABS4062-contig_7; Figure 6) is questionable, as the borders of the prophage regions appear to be truncated by the ends of the contigs in which they were found. However, phylogenetic analysis allows us to propose a classification for these phages. The phages integrated into contig_6 of the chromosome of isolate ABS30421 can be confidently classified within the *Vieuvirus* genus, which includes temperate siphoviruses with genomes of approximately 45–50 kbp [51].

BLAST searches using the MCP and TLS found in the prophage located in contig_7 of isolate ABS82146 did not reveal homologs of these proteins among phages infecting *Acinetobacter* bacteria. Instead, they pointed to the *Ralstonia* phage Firinga of the *Firingavirus* genus and the *Sodalis* phage phiSG1 (unclassified) as closely related phages. This suggests that prophage ABS82146-contig_7 may represent a novel group of temperate phages infecting *A. baumannii*, potentially classifiable within a new taxon at the rank of genus or higher.

Similarly, BLAST searches using the MCP and TLS of the prophage found in contig_7 of isolate ABS4062 also pointed to the closest homologs from two different phages, both infecting *A. baumannii*. Although the MCP homolog was found in the genome of *Acinetobacter* phage Ab105-2phi, which is attributed to the *Vieuvirus* genus in GenBank, this classification appears to be erroneous. Unlike the case of prophage ABS30421-contig_6, the MCPs of both the prophage ABS4062-contig_7 and *Acinetobacter* phage Ab105-2phi do not demonstrate homology with phages classified within the *Vieuvirus* genus by the International Committee on Taxonomy of Viruses (ICTV). Intergenomic similarity calculations using VIRIDIC revealed that *Acinetobacter* phage Ab105-2phi exhibits significant, but insufficient for classification within the genus *Vieuvirus*, similarity scores of 35.5% and 37.6% to the officially classified *Vieuvirus* phages B1251 and R3177, respectively. Prophage ABS4062–contig_7 demonstrates even lower intergenomic similarity, with scores of 17.8% to Vieuvirus B1251, 15.0% to Vieuvirus R3177, and 18.2% to phage Ab105-2phi.

#### 3.3.3. General Characterization of Prophage Regions in *K. pneumoniae* Genomes

**Figure 7:**
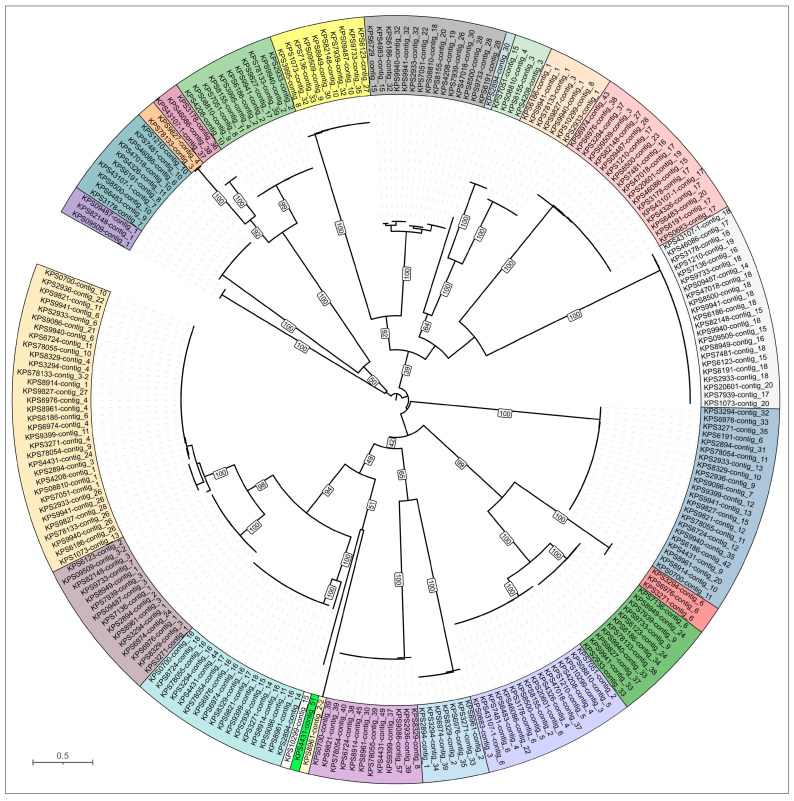
Maximum-likelihood phylogenetic tree based on amino acid sequences of MCP encoded in *K. pneumoniae* prophage regions and related phages. Distinct clusters from which representative prophage regions were analyzed are indicated by different colors. Bootstrap values are shown near the nodes. The scale bar indicates 0.5 estimated substitutions per site, and the tree is midpoint-rooted.

To comprehensively investigate the prevalence of prophages in *Klebsiella pneumoniae*, we employed a similar bioinformatics approach. The genomes of 52 *K. pneumoniae* isolates were analyzed using PHASTEST to identify potential prophage regions and HHblits to detect the presence of MCP and TLS. This integrated search revealed a significant number of putative prophage regions across the dataset. In total, 252 distinct regions with prophage characteristics were identified. Of these, PHASTEST identified 235 prophage regions, while additional searches identified 17. Therefore, the average number of putative prophages per *K. pneumoniae* genome was approximately 4.8. This number is somewhat lower than the average observed in our study of *A. baumannii* isolates, where we found approximately six prophage-derived regions per genome. One prophage region lacked a major capsid protein gene and likely represented the remains of a domesticated prophage. The remaining 246 prophages were then grouped via phylogenetic analysis of their MCP sequences (Figure 7). This analysis identified 24 distinct clusters, from which a single representative prophage was selected for detailed annotation and subsequent analysis (Table 3). Twenty-four prophage regions representing different groups in the phylogenetic trees were annotated using BLAST and HMM-based methods (Supplementary File S2).

A BLAST search of the phage GenBank database using the MCP sequences identified related phages for all representative putative prophages (E-value threshold 10^−5^). The majority of prophages (19 out of 24) exhibited close or near-identical similarity to previously isolated phages infecting *Klebsiella* bacteria (amino acid pairwise identity ≥ 95%). Three representative MCPs exhibited a close relationship between their corresponding prophage regions and phages infecting other members of the order Enterobacterales. Two remaining representative MCPs demonstrated similarity most likely to the MCPs of phages infecting other groups of bacteria, including *Stenotrophomonas maltophilia* (the MCP from the previously isolated, unclassified phage *Stenotrophomonas* vB_SmeS_BUCT709 and the MCP from a prophage region located in contig_3 of *K. pneumoniae* isolate KPS78133 shared >99% identity) and, surprisingly, a *Comamonadaceae* bacterium related to *Curvibacter delicatus* (*Curvibacter* phage P26059A [52] and the prophage region located in contig_7 of *K. pneumoniae* isolate KPS3178). However, the latter prophage MCP exhibited only a low degree of similarity to the MCP sequence of the *Curvibacter* phage P26059A (pairwise identity 26%) and other phage MCP sequences, suggesting only a distant relationship between the putative prophage KPS3178_contig_7 and known isolated phages. Furthermore, the prophage region KPS3178_contig_7 had the highest percentage of orphan genes (81%). However, these results may be due to the degradation of genes within the prophage, which was domesticated comparatively long ago.

**Figure 8:**
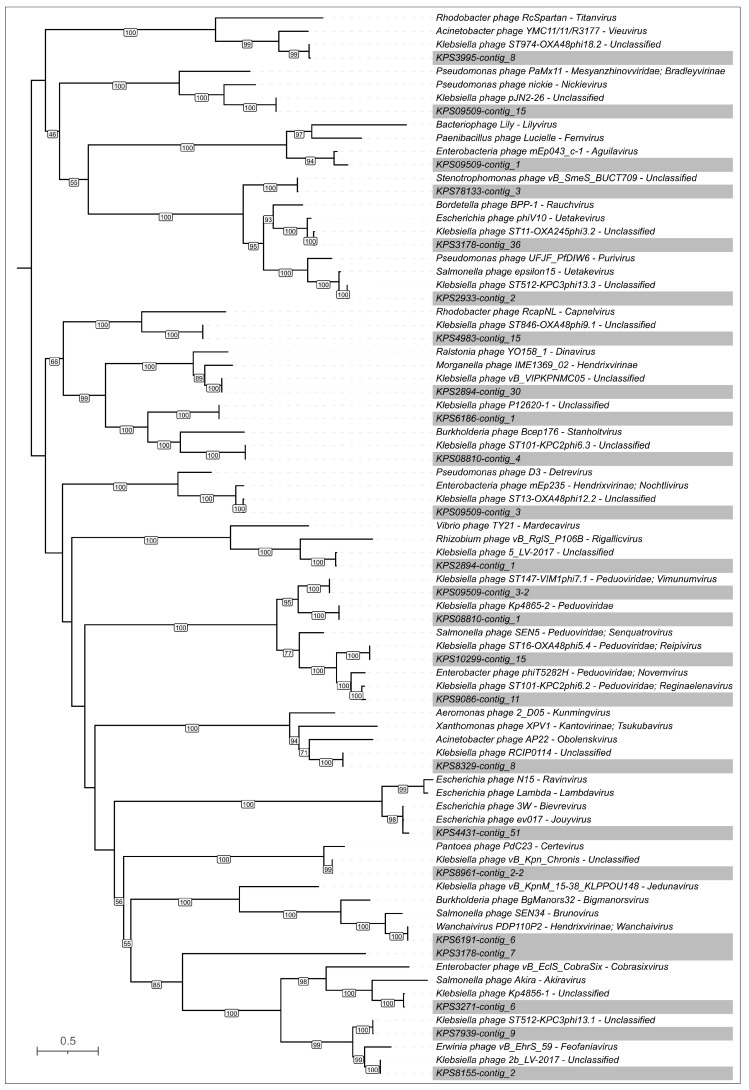
Maximum-likelihood phylogenetic tree based on amino acid sequences of MCP encoded in *K. pneumoniae* prophage regions and related phages. NCBI taxonomy is shown to the right of phage names. Bootstrap values are shown near the nodes. The scale bar indicates 0.5 estimated substitutions per site, and the tree is midpoint-rooted. Representative prophage regions are colored gray.

The subsequent phylogenetic analysis (Figure 8) employed the MCP sequences identified using the aforementioned BLAST search, along with the MCP sequences of representative prophages. The sequences used in the tree were refined to include the highest-scoring hits and the most closely classified phage groups. This analysis suggested the classification of four putative prophages (labeled KPS09509-contig_3-2, KPS08810-contig_1, KPS10299-contig_15, and KPS9086-contig_11) within the *Peduoviridae* family and a putative prophage KPS6191-contig_6 within the *Hendrixvirinae* subfamily. Notably, most of the closely related phages infecting *Klebsiella* bacteria are unclassified, indicating that phage taxonomy is struggling to keep pace with the expanding volume of genomic data. However, most related non-*Klebsiella* phages belong to taxa comprising temperate phages, including those classified within the *Peduoviridae* family, *Hendrixvirinae* subfamily, *Aguilavirus*, *Bievrevirus*, *Jouyvirus*, *Lambdavirus*, *Purivirus*, *Uetakevirus*, *Vieuvirus*, and other genera. Two prophage regions showed distinct relatedness to phages infecting *A. baumannii*, including the temperate *Acinetobacter* phage YMC11/11/R3177 of the *Vieuvirus* genus [53] and the lytic phage AP22 of the *Obolenskvirus* genus [54]. Notably, lytic phages of the *Obolenskvirus* genus have been suggested to be evolutionarily related to phages adopting a temperate lifestyle [55].

The morphology of putative phages that gave rise to the analyzed representative prophage regions was inferred based on the presence of characteristic encoded proteins, according to HMM analysis. Specifically, tail sheath proteins indicated myoviruses, T7-like tail tubular proteins and internal virion proteins indicated podoviruses, and the morphology of phages with homologous structural proteins was also considered. Half of the representative prophages (twelve regions) are likely to represent siphoviruses, seven regions appear to have originated from the integration of myoviruses, four represent podoviruses, and the region KPS3178_contig_7, apparently representing a comparatively distant phage group, might belong to siphoviruses, although a more definitive determination of morphology requires further study. Analysis of genetic content revealed signs of domestication in some prophages, a process accompanied by gene degradation and loss as well as unexpected insertions within genetic modules. For example, gene prediction results indicated that the prophage region in contig_51 of *K. pneumoniae* isolate KPS4431 contains a split major capsid protein, similar to that observed in *A. baumannii* isolate ABS4984 (the region located in contig_9), and inclusions of oppositely oriented genes within structural modules.

**Figure 9:**
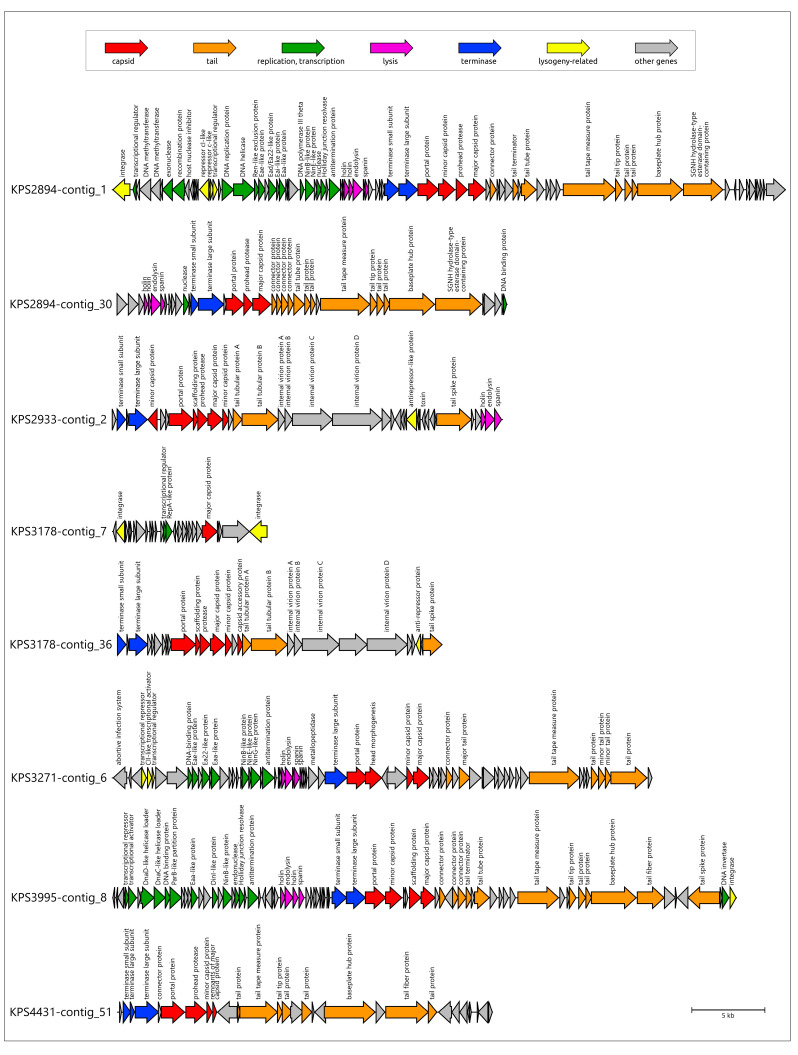

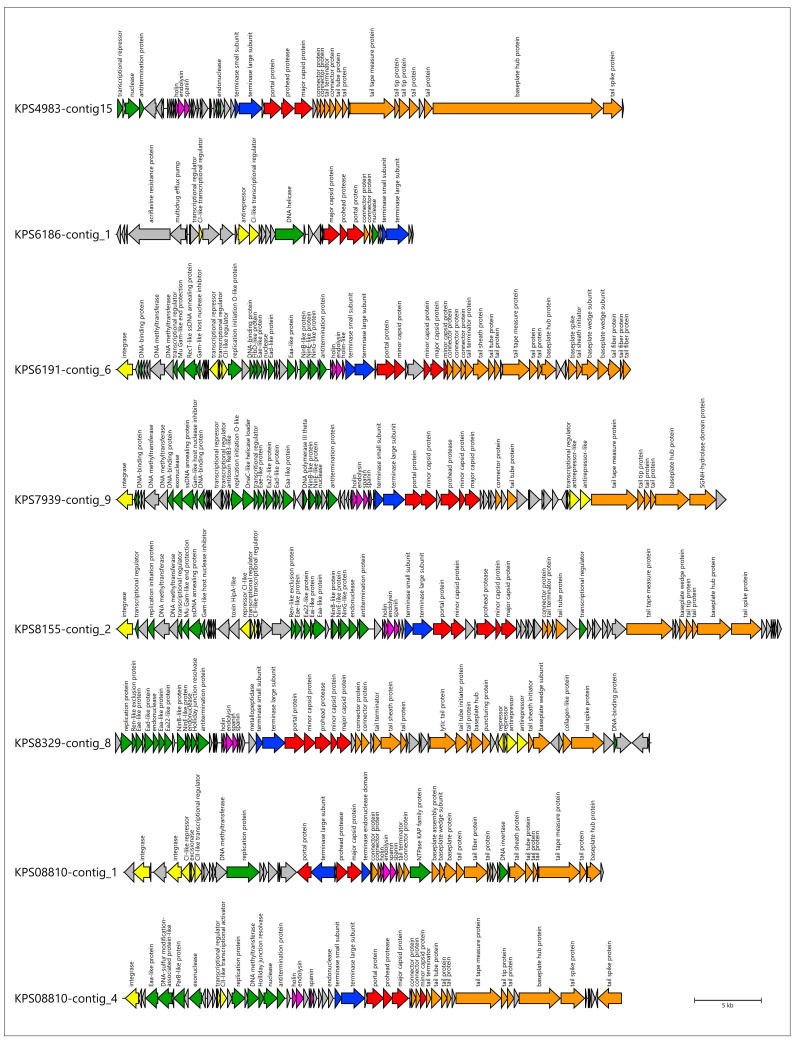

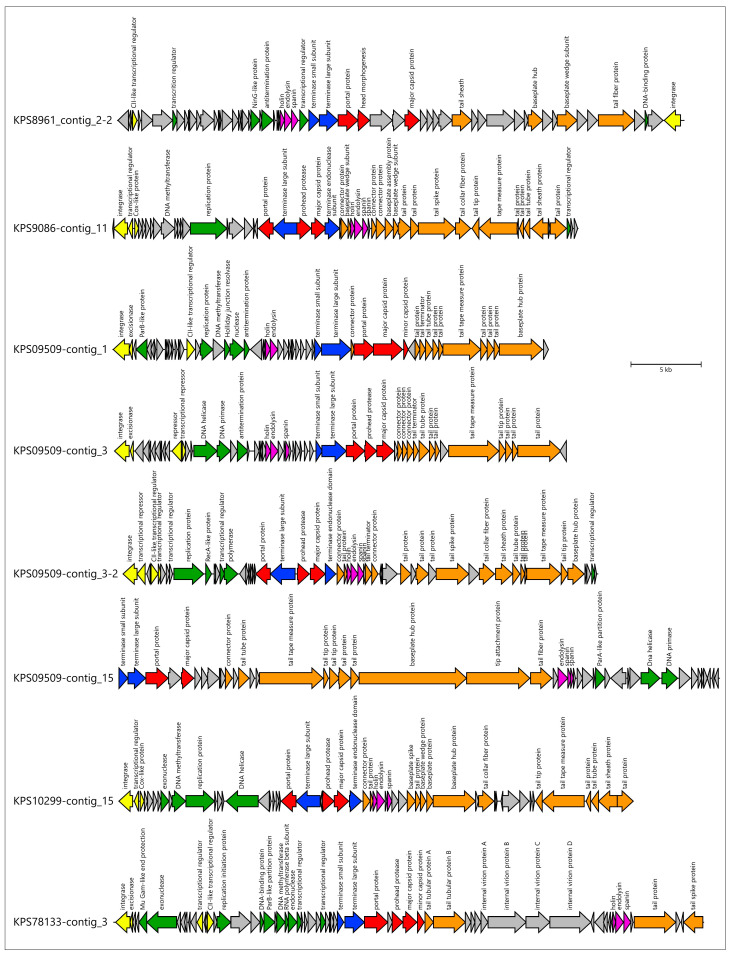
Genetic maps of prophage regions revealed in the genomes of different *K. pneumoniae* isolates. Gene annotations and predicted functions are indicated by labels and a legend. Arrows show the direction of transcription for each gene. The scale bar represents the length of the nucleotide sequence.

However, most regions not truncated by contig borders possess all functional modules characteristic of phage genomes. In several prophage regions (KPS2894-contig_1, KPS3271-contig_6, KPS3995-contig_8, KPS6191-contig_6, KPS7939-contig_9, KPS8155-contig_2, KPS8329-contig_8, KPS08810-contig_4, KPS09509-contig_3, KPS09509-contig_15, KPS78133-contig_3), gene function prediction indicated the presence of a comparatively sophisticated replication, recombination, and regulation machinery (Figure 9). The functional content of this machinery is reminiscent of that found in *Escherichia* phage λ and other temperate phages. This apparatus presumably includes λ-like replication proteins O and P [56], proteins of the *ninR* region [57], and Ea22 and other early gene-associated proteins [58]. The composition and architecture of this module appear similar in both myovirus-related and siphovirus-like prophage regions. Furthermore, the identified genes and composition of the genetic modules associated with the lysogeny decision also share similarity with their counterparts from phage λ and other temperate phages, including those of *Lederbergviruses*, *Detreviruses*, etc. As expected, structural modules of putative prophages are generally similar within the same morphotype; however, differences can be observed in genes encoding predicted RBPs. Notably, unlike other prophage regions, the putative baseplate hub protein (BHP) of KPS4983-contig_15 is exceptionally large (4234 amino acids) and contains multiple domains supposedly involved in carbohydrate binding, similar to the BHP of region KPS09509-contig_15 (AlphaFold models are shown in Supplementary Figure S3). Importantly, most representative prophage regions apparently contain TSP and TFP, and two prophage regions (KPS3995-contig_8 and KPS08810-contig_4) contain sets of two distinct TSPs. Finally, the lysis modules identified in prophage regions contain genes encoding holin, endolysin, and spanin, but several prophages apparently contain two holin-like genes that can encode both holin and anti-holin [59].

### 3.4. Analysis of Identification of PTDEs in A. baumannii and K. pneumoniae Genomes

A search for PTDEs in the analyzed bacterial genomes was conducted according to the aforementioned protocol (Figure 3), which included BLAST and HHblits searches using all predicted bacterial proteins, along with a manual inspection of proteins encoded within identified prophage regions and AlphaFold modeling and consequent DALI search using the structural models of modeled putative RBPs within the representative prophage regions. This search revealed 6 PTDEs in the genomes of 15 *A. baumannii* isolates and 133 PTDEs in the genomes of 52 *K. pneumoniae* isolates. The *A. baumannii* PTDEs contained enzymatic domains exclusively belonging to esterases of the SGNH-hydrolase protein family, whereas the *K. pneumoniae* PTDEs contained enzymatic domains similar to both esterases and depolymerases, including various glycosidases and lyases.

**Figure 10:**
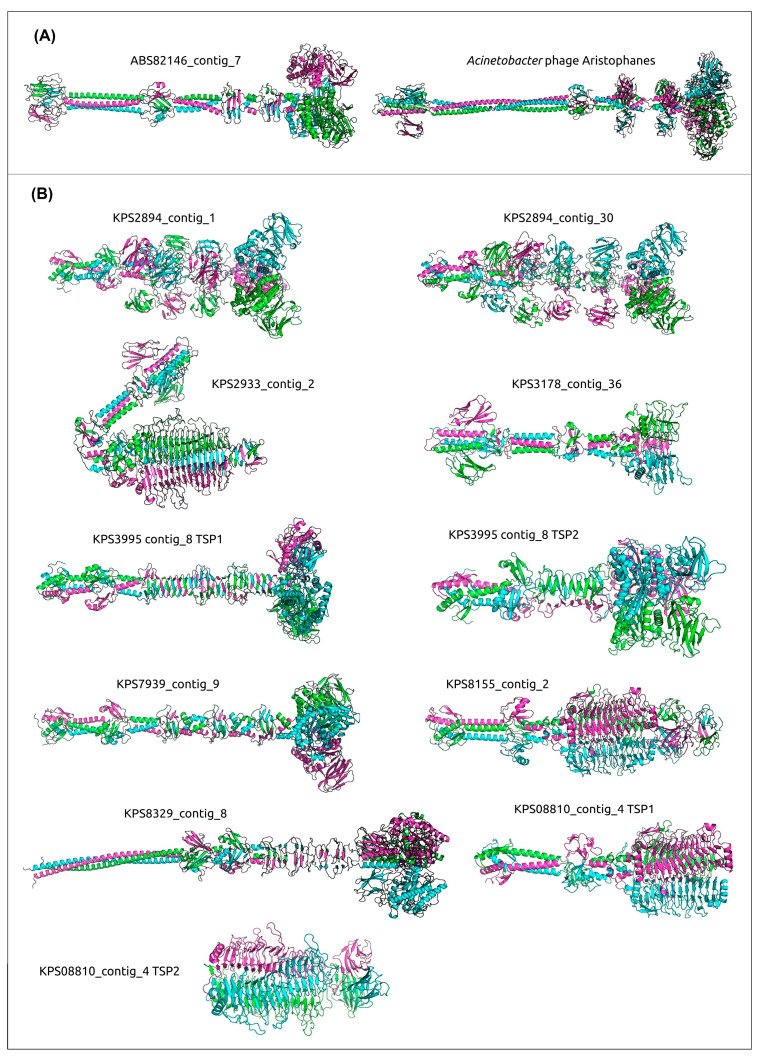
Predicted structures of putative trimeric *A. baumannii* and *K. pneumoniae* prophage-derived tailspike depolymerases and esterases, modeled using AlphaFold. (**A**) Structural architecture comparison of the tailspike esterase ABS82146_contig_7 and the TSP of the *Acinetobacter* phage Aristophanes. (**B**) Predicted structures of PTDE identified in *K. pneumoniae* prophage regions. Different monomers are distinctly colored.

All genes encoding *A. baumannii* prophage-derived PTDEs were identified exclusively within the genomes of six isolates (ABS09481, ABS09493, ABS82612, ABS82603, ABS82146, ABS09593) belonging to the same cluster in the heatmap and the phylogenetic tree (Figure 1A,B) and carrying the same KL3. Two of these genes were identified within two representative prophage genomic regions of *A. baumannii*, specifically in isolates ABS82146_contig_7 and ABS09481_contig_9 (Figure 6). These PTDEs exhibit strong similarity (HHpred probability near 100%) to various O-acetylesterases, including sialate O-acetylesterases from *Canis familiaris* (PDB #8F9O [60]) and *Xanthomonas axonopodis* pv. *citri* (PDB #7KMM [61]). These two esterases share a moderate level of similarity with each other (pairwise identity, PI 57%). In contrast, the MCPs of these putative prophages exhibit significantly lower similarity (PI 14%), indicating only a distant relationship between the phages from which the prophage regions originated and suggesting the occurrence of genetic exchange involving the PTDE genes of these prophages. It is plausible that the corresponding phages utilize capsular polysaccharides as receptors, removing the O-acetyl group from the capsular polysaccharide, as previously described for the *Acinetobacter* phage Aristophanes [62]. AlphaFold predicted a similar structural architecture for putative trimeric TSPs from the predicted prophages ABS82146_contig_7 and ABS09481_contig_9 and the TSP of the phage Aristophanes (Figure 10).

Analysis of 24 representative prophage regions of *K. pneumoniae* indicated the presence of sixteen genes potentially encoding PTDEs. Several PTDEs exhibited notable similarity to each other (PI > 70%, exceeding the PI values of the corresponding MCPs); these included the following three pairs: KPS2933-contig_2 (the AlphaFold models are presented in Figure 10) and KPS78133-contig_3 (Pair 1), KPS8155-contig_2 and KPS08810-contig_4-TSP1 (Pair 2), and KPS8329-contig_8 and KPS8961_contig_2-2 (Pair 3). Proteins from pairs 1 and 2 likely represent tailspike depolymerases and, according to the HHpred search results, are similar to various phage- or bacterial-derived depolymerases, including glycosidases and lyases. Proteins from pair 3 contain SGNH-hydrolase domains and exhibit strong similarity (HHpred probability exceeding 99%) to various O-acetylesterases. The corresponding *K. pneumoniae* isolates within pairs 1 and 2 shared the same KL type (107 and 17, respectively), whereas the isolates from pair 3 exhibited different KL types (39 for KPS8329 and 2 for KPS8961). While the first two cases can be explained by horizontal transfer involving gene modules encoding phage RBPs, a common feature of phages infecting *Klebsiella* and other bacteria [63], the latter case is intriguing and warrants further investigation. ANI clustering placed both isolates, KPS8329 and KPS8961, within the same major cluster (lower-right in the clustered heatmap in Figure 2A), but phylogenetic analysis of a concatenated alignment of marker genes (Figure 2B) assigned them to distinct clades. Moreover, the distribution of KL types of *K. pneumoniae* was not uniquely determined by position in either the phylogenetic tree or the ANI heatmap; the same clades or clusters contained representatives of diverse capsular types. This observation likely reflects genetic exchange between bacteria, a process also involving genetic loci responsible for capsular polysaccharide biosynthesis [64]. It is possible that the integration of a phage leading to the appearance of the prophage region KPS8329-contig_8 occurred before a chromosomal recombination event resulting in switching of the KL type. Furthermore, we cannot rule out the possibility that the putative RBPs of the phages represented by proteins of pair 3 utilize a receptor other than capsular polysaccharide and degrade it through O-deacetylation.

In general, SGNH-hydrolase domain-containing proteins are prevalent among predicted PTDEs within representative prophage regions. According to HHpred results using the amino acid sequences and DALI searches using the modeled structures, these domains were found in prophage regions within the sequences of eight out of ten remaining representative prophages. Interestingly, both PTDEs from isolate KPS2894, found within contig_1 and contig_30, contain multiple Ig-like domains (Figure 10), which could enhance binding to carbohydrates [65] and are found in various phage structural proteins, including MCP [66], tail tube [67], and tail sheath [68] proteins. Additionally, both PTDEs shared similarity (HHpred probability > 99.9% and DALI Z-score > 30) with O-acetylesterases from *Neisseria meningitidis* (PDB #4K7J [69]) and *Campylobacter jejuni* (PDB #8GKD). The PTDE containing an SGNH-hydrolase domain and similar to several O-acetylesterases, including the aforementioned one from *Neisseria meningitidis*, is encoded within the prophage region KPS3995-contig_8. This PTDE (TSP1) constitutes one of two TSPs encoded in this prophage region. The other TSP of KPS3995-contig_8 (TSP2) contains two distinct SGNH-hydrolase domains, in the middle and C-terminal regions, which are more similar to different sets of acylhydrolases, according to HHpred and DALI. This may broaden the host specificity of the original phage. The putative TSP from the region located in contig_9 of isolate KPS7939, like the aforementioned TSPs from contig_1 and contig_30 of isolate KPS2894, is similar to O-acetylesterases from *Neisseria meningitidis* (PDB #4K7J) and *Campylobacter jejuni* (PDB #8GKD). Also, PTDEs located within prophage regions KPS8961_contig_2-2 and KPS09509-contig_3-2 exhibited resemblance to O-acetylesterases from *Canis familiaris* (PDB #8F9O [59]) and *Xanthomonas axonopodis* pv. *citri* (PDB #7KMM [60]), similar to the case mentioned above for prophages of *A. baumannii*. Finally, the SGNH-hydrolase domain phage tail protein encoded in the prophage region KPS9086-contig_11 exhibited a higher resemblance with hydrolases from *Lactobacillus plantarum* (PDB code #3DC7), *Veillonella parvula* (PDB code #4RW0), and from *Collinsella aerofaciens* (PDB code # 7BXD) (HHpred probability > 99.6%).

The remaining two modeled structures of the predicted TSPs from representative prophage regions, particularly KPS3178-contig_36, were predicted to exhibit an architecture distinct from the acylhydrolase-like domain-containing PTDEs described above. The trimeric structure featured a triple β-helical fold formed by the polypeptide chains, a characteristic motif found in many phage tail RBPs, including those from *K. pneumoniae*-infecting phages [70,71,72]. Alongside the structures described above, located within the prophage regions KPS2933-contig_2, KPS78133-contig_3, KPS8155-contig_2, and TSP1 encoded by KPS08810-contig_4, this group of proteins includes TSP2 encoded by KPS08810-contig_4 and the PTDE encoded by KPS3178-contig_36. The gene encoding the TSP within the prophage region KPS3178-contig_36 appears to be truncated at its 3′ end by the contig’s border. Nevertheless, HHpred and DALI analyses indicated that this TSP, in addition to the N-terminus, which shares similarity with the virion-binding domains of other tail RBPs, contains triple β-helical regions resembling the catalytic domains of various glycosidases, such as glycosidases from *Escherichia* phage CBA120 [73] (PDB #6NW9, HHpred probability 99.5%, DALI Z-score 14.5) and *Klebsiella* phage KP32 [72] (PDB #6TKU, HHpred probability 99.2%, DALI Z-score 6.9), among others. This TSP also shows similarity to pectin lyases, including those from *Klebsiella* phage SH-Kp 152410 [70] (PDB #8X8M, HHpred probability 99.2%) and *Geobacillus* phage E2 [74] (PDB #7CHU, HHpred probability 98.6%, DALI Z-score 20.9). Furthermore, the PTDE from contig_4 of isolate KPS08810 assembly, representing TSP2, is also truncated and contains a depolymerase domain similar to various glycosidases and lyases, including those mentioned during the previous analysis of TSP from the prophage region KPS3178-contig_36.

## 4. Discussion

*A. baumannii* and *K. pneumoniae* isolates, studied in this work, were obtained from the blood of intensive care unit patients of the multidisciplinary scientific and practical center of emergency medicine from January to September 2024. All isolates were divided into several MLST (according to the Pasteur scheme) and KL groups. Among XDR *A. baumannii* isolates (n = 15), more than a third of the isolates were found to belong to ST2 (n = 6), and three isolates were assigned to ST19, which are the members of the most disseminated high-risk *A. baumannii* global clone (GC) 1 and GC2, respectively [75,76], with ST2 being the predominant clone identified worldwide [77,78]. More than a third of the *A. baumannii* isolates were assigned to ST78 (n = 6), which was also found and spread in different countries [79,80,81]. Among the XDR and PDR *K. pneumoniae* isolates (n = 52), seven isolates belong to ST147, six isolates—to ST512, four—to ST101, and two—to ST23, which are assigned to the well-characterized disseminated worldwide high-risk clonal group (CG) CG147 [82], CG258 [83], CG101 [84], and CG23 [85], respectively. The majority of *K. pneumoniae* isolates belonged to ST395 (n = 16) and ST39 (n = 14), which are common genetic lines identified in Russia [86]. KLs identified in the genomes of *A. baumannii* isolates include clusters such as KL3, KL49, and KL17, which were bioinformatically predicted in 13.3%, 3.7%, and 2.9% of 8994 *A. baumannii* genome assemblies deposited to the NCBI database, respectively [13]. The *K. pneumoniae* genomes carry 12 different KLs, including KL1, KL2, KL23, and KL64, which are responsible for the synthesis of the CPSs of the most commonly spread K types [87,88].

Using genomic sequence analysis, *A. baumannii* and *K. pneumoniae* isolates were clustered into distinct groups based on ANI calculations and phylogenetic analysis using concatenated alignments of conserved proteins. Notably, a distinct correlation was observed, albeit not absolute, between the KL- and MLST types of the *A. baumannii* and *K. pneumoniae* isolates and their positions in the GTDB-Tk phylogenetic trees or the ANI-based clustered heatmaps.

The further identification of prophage regions and prophage-derived PTDEs in the genomes of the isolates was carried out using the workflow protocol, which was chosen in such a way as to take into account the complexities of bacterial evolution and genomic data acquisition. Given the high frequency of recombination in bacterial genomes and the potential fragmentation of prophage region sequences during NGS sequencing, a comprehensive search across all genes in the bacterial genome, rather than solely within bioinformatically predicted prophage-derived loci, appears justified. Moreover, any bioinformatics method that relies on homology or similarity searches against databases is inherently limited by the size and quality of such databases. This limitation likely contributed to the lower number of prophage-derived regions identified by PHASTEST compared with combined searches involving complete screening of all predicted bacterial proteins using HHblits and extensive databases. Notably, the additional search for prophages beyond PHASTEST yielded nearly half the sequences for *A. baumannii*, but only ~7% for *K. pneumoniae*, possibly reflecting the differing representation of these organisms and their related phages in the PHASTEST databases. In fact, maintaining up-to-date bioinformatic databases amidst the growing volume of data and new discoveries presents significant challenges. The recent discovery of filamentous phages infecting *A. baumannii* [89] exemplifies this, and it is unsurprising that inovirus-like proteins were identified in the analyzed genomes through comprehensive searches against all bacterial proteins. Indeed, our understanding of filamentous phage diversity has expanded significantly in recent years with the identification of novel phage groups [89,90,91,92]. Although these phages play a crucial role in bacterial infection processes [93], their specific influence on *A. baumannii* virulence and pathogenicity remains understudied and warrants a detailed investigation.

The total number of prophages identified in the genomes of the analyzed *A. baumannii* isolates (averaging 6.00 prophages per genome) generally aligns with previously published data (6.53 prophages per genome in [94] and 6.01 in [95]). Interestingly, while most of the identified prophage regions exhibit close relationships to previously isolated temperate *A. baumannii*-infecting phages, the presence of sequences related only to distantly related phages underscores the existing gaps in our knowledge regarding *A. baumannii*-infecting phages. It appears that novel phage groups can still be discovered even within the generally well-studied phages infecting *K. pneumoniae* (*Klebsiella*-infecting phages are represented by more than one thousand sequences in the GenBank database). However, the majority of prophage regions identified in the analyzed *K. pneumoniae* genomes were closely related to previously published sequences. Moreover, the average number of prophage regions (averaging 4.8 per genome) was within the range of previously published results (3.7 [96], 5.4 [97], and 9 prophages [98]).

Our analysis identified several proteins of apparent phage origin that exhibited similarity to phage-derived depolymerases and esterases. However, this similarity occurred frequently at the nonenzymatic domain level. This highlights the necessity for careful analysis of the domain architecture of candidate PTDEs and the significant potential of modern structural modeling methods, such as AlphaFold, for characterizing putative PTDEs. Notably, within both *A. baumannii* and *K. pneumoniae*, the predicted prophage regions preferentially contained PTDEs belonging to esterases rather than depolymerases, but this observation could be a characteristic feature of the isolates circulating within a certain hospital. Recognizing that PTDEs are proteins specifically degrading or modifying CPSs, key virulence factors of bacterial cells, identification, characterization of such enzymes, and determination of their substrate specificity will significantly contribute to the development of effective strategies aimed at controlling the spread of *A. baumannii* and *K. pneumoniae* strains belonging to different K types.

## Figures and Tables

**Table 1:** General characterization of *A. baumannii* and *K. pneumoniae* isolates.

No.	Strain Designation	Data of Isolation	Source of Isolation	GenBank Accession Number	KL	MLST	SCPM-Obolensk Accession Number	Antibiotic Resistance Phenotype
***A. baumannii***								
1	ABS4984	07.02.2024	blood	JBMMJM000000000	17	19	B-15821	XDR
2	ABS4062	12.02.2024	blood	JBMMJL000000000	17	19	B-15822	XDR
3	ANS7072 *	23.04.2024	blood	JBMMJD000000000	49	2	B-16457	XDR
4	ABS09493	02.05.2024	blood	JBMMJJ000000000	3	78	B-16451	XDR
5	ABS09481	02.05.2024	blood	JBMMJI000000000	3	78	B-16452	XDR
6	ABS09593	03.05.2024	blood	JBMMJF000000000	3	78	B-16455	XDR
7	ABS78057	04.05.2024	blood	JBMMJG000000000	17	19	B-16454	XDR
8	ABS82612	13.05.2024	blood	JBMMJK000000000	3	78	B-16450	XDR
9	ABS82603	13.05.2024	blood	JBMMJH000000000	3	78	B-16453	XDR
10	ABS82146	13.05.2024	blood	JBMMJE000000000	3	78	B-16456	XDR
11	ABS30421	20.05.2024	blood	JBMMHS000000000	235	2	B-17190	XDR
12	ABS2640	06.06.2024	blood	JBMMHQ000000000	235	2	B-17192	XDR
13	ABS7948	07.06.2024	blood	JBMMHR000000000	235	2	B-17191	XDR
14	ABS1720	06.07.2024	blood	JBMMHO000000000	235	2	B-17194	XDR
15	ABS8964	29.07.2024	blood	JBMMHP000000000	235	2	B-17193	XDR
***K. pneumoniae***								
1	KPS0683	09.01.2024	blood	JBMMJP000000000	23	39	B-15818	PDR
2	KPS0700	10.01.2024	blood	JBMMJO000000000	48	395	B-15819	PDR
3	KPS4326	15.01.2024	blood	JBMMJN000000000	23	39	B-15820	PDR
4	KPS9399	26.01.2024	blood	JBMMJC000000000	48	395	B-16458	PDR
5	KPS9821	29.01.2024	blood	JBMMKC000000000	48	395	B-15805	PDR
6	KPS4208	30.01.2024	blood	JBMMJZ000000000	17	101	B-15808	XDR
7	KPS9827	31.01.2024	blood	JBMMKA000000000	107	512	B-15807	XDR
8	KPS6724	01.02.2024	blood	JBMMKB000000000	48	395	B-15806	PDR
9	KPS7051	01.02.2024	blood	JBMMIL000000000	17	101	B-16476	XDR
10	KPS6729	03.02.2024	blood	JBMMJY000000000	1	23	B-15809	PDR
11	KPS4983	05.02.2024	blood	JBMMJX000000000	1	23	B-15810	PDR
12	KPS3995	12.02.2024	blood	JBMMJW000000000	57	218	B-15811	XDR
13	KPS2933	17.02.2024	blood	JBMMJV000000000	107	512	B-15812	XDR
14	KPS7481	17.02.2024	blood	JBMMJU000000000	23	39	B-15813	XDR
15	KPS1210	19.02.2024	blood	JBMMJR000000000	23	39	B-15816	XDR
16	KPS7939	29.02.2024	blood	JBMMJT000000000	64	39	B-15814	XDR
17	KPS9733	04.03.2024	blood	JBMMJS000000000	64	147	B-15815	XDR
18	KPS8329	06.03.2024	blood	JBMMJQ000000000	39	395	B-15817	XDR
19	KPS6974	16.03.2024	blood	JBMMIP000000000	2	395	B-16471	XDR
20	KPS6976	25.03.2024	blood	JBMMIQ000000000	2	395	B-16470	XDR
21	KPS6483	30.03.2024	blood	JBMMIJ000000000	23	39	B-16478	XDR
22	KPS09487	02.04.2024	blood	JBMMIT000000000	20	147	B-16467	XDR
23	KPS9086	04.04.2024	blood	JBMMII000000000	48	395	B-16479	XDR
24	KPS1073	11.04.2024	blood	JBMMIO000000000	112	15	B-16472	XDR
25	KPS2936	11.04.2024	blood	JBMMIM000000000	48	395	B-16475	XDR
26	KPS3294	15.04.2024	blood	JBMMIR000000000	2	395	B-16469	XDR
27	KPS3178	15.04.2024	blood	JBMMIN000000000	23	39	B-16473	XDR
28	KPS3271	15.04.2024	blood	JBMMIK000000000	2	395	B-16477	XDR
29	KPS82148	13.05.2024	blood	JBMMJA000000000	20	147	B-16460	XDR
30	KPS47018	22.04.2024	blood	JBMMIZ000000000	23	39	B-16461	XDR
31	KPS43107-1	22.04.2024	blood	JBMMIV000000000	23	39	B-16465	XDR
32	KPS46086	24.04.2024	blood	JBMMJB000000000	23	39	B-16459	XDR
33	KPS78055	02.05.2024	blood	JBMMIY000000000	48	395	B-16462	XDR
34	KPS09509	02.05.2024	blood	JBMMIX000000000	20	147	B-16463	XDR
35	KPS78054	02.05.2024	blood	JBMMIS000000000	48	395	B-16468	XDR
36	KPS78133	03.05.2024	blood	JBMMIU000000000	107	512	B-16466	PDR
37	KPS08810	06.05.2024	blood	JBMMIW000000000	17	101	B-16464	XDR
38	KPS20601	08.07.2024	blood	JBMMIH000000000	23	39	B-17173	XDR
39	KPS2894	08.07.2024	blood	JBMMIG000000000	39	39	B-17174	XDR
40	KPS8961	29.07.2024	blood	JBMMIF000000000	2	395	B-17175	XDR
41	KPS9940	05.08.2024	blood	JBMMIE000000000	107	512	B-17177	PDR
42	KPS9941	05.08.2024	blood	JBMMID000000000	107	512	B-17179	PDR
43	KPS6123	12.08.2024	blood	JBMMIB000000000	64	147	B-17181	XDR
44	KPS10299	12.08.2024	blood	JBMMIC000000000	102	307	B-17180	XDR
45	KPS8949	15.08.2024	blood	JBMMIA000000000	64	147	B-17182	XDR
46	KPS7136	19.08.2024	blood	JBMMHZ000000000	64	147	B-17183	XDR
47	KPS8914	19.08.2024	blood	JBMMHY000000000	48	395	B-17184	XDR
48	KPS8155	10.09.2024	blood	JBMMHX000000000	17	101	B-17185	XDR
49	KPS8500	16.09.2024	blood	JBMMHW000000000	23	39	B-17186	XDR
50	KPS6191	16.09.2024	blood	JBMMHV000000000	64	39	B-17187	XDR
51	KPS6186	16.09.2024	blood	JBMMHU000000000	107	512	B-17188	XDR
52	KPS4431	17.09.2024	blood	JBMMHT000000000	48	395	B-17189	XDR

* the isolate ANS7072 was identified as *Acinetobacter nosocomialis* using a MALDI-TOF Biotyper system (Bruker Daltonics, Bremen, Germany). Therefore, it was initially designated with “ANS” prefix. Then, after analyzing the WGS data, this isolate was assigned to the species *A. baumannii*.

**Table 2:** General characterization of prophage regions in *A. baumannii* isolates.

Designation of Prophage Region	Size, bp	% GC	Related GenBank Phage Sequence (Taxonomy)
ABS09481-contig_18	66,919	37.0%	*Acinetobacter* phage vB_AbaM_ABMM1 (Unclassified)
ABS09481-contig_9	35,087	39.4%	*Moraxella* phage Mcat9 (Unclassified)
ABS30421-contig_6	25,324	39.8%	*Acinetobacter* phage vB_AbaS_Eva (*Vieuvirus*)
ABS4062-contig_7	48,276	36.7%	*Acinetobacter* phage Ab105-2phi (*Vieuvirus*)
ABS4984-contig_9	33,074	35.4%	*Acinetobacter* phage ZaA-2018b (Unclassified)
ABS82146-contig_7	25,454	41.5%	*Ralstonia* phage Firinga (*Firingavirus*)
ABS8964-contig_4	38,416	39.9%	*Acinetobacter* phage fEg-Aba01 (Unclassified)

**Table 3:** General characterization of prophage regions in *K. pneumoniae* isolates.

Designation of Prophage Region	Size, bp	% GC	Related GenBank Phage Sequence (Taxonomy)
KPS08810-contig_1	36,056	50.8%	*Klebsiella* phage Kp4865-2 (*Peduoviridae*)
KPS08810-contig_4	37,471	50.6%	*Klebsiella* phage Kp4865-2 (*Peduoviridae*)
KPS09509-contig_1	31,076	50.2%	*Enterobacteria* phage mEp043_c-1 (*Aguilavirus*)
KPS09509-contig_15	42,861	50.7%	*Klebsiella* phage pJN2-26 (Unclassified)
KPS09509-contig_3	32,211	50.8%	*Klebsiella* phage ST13-OXA48phi12.2 (Unclassified)
KPS09509-contig_3-2	33,882	51.8%	*Klebsiella* phage ST147-VIM1phi7.1 (*Peduoviridae; Vimunumvirus*)
KPS10299-contig_15	36,702	49.8%	*Klebsiella* phage ST16-OXA48phi5.4 (*Peduoviridae; Reipivirus*)
KPS2894-contig_1	46,173	52.7%	*Klebsiella* phage 5_LV-2017 (Unclassified)
KPS2894-contig_30	26,753	51.1%	*Klebsiella* phage vB_VIPKPNMC05 (Unclassified)
KPS2933-contig_2	26,795	51.6%	*Klebsiella* phage ST512-KPC3phi13.3 (Unclassified)
KPS3178-contig_36	22,258	55.2%	*Klebsiella* phage ST11-OXA245phi3.2 (Unclassified)
KPS3178-contig_7	10,620	39.7%	*Curvibacter* phage P26059A (Unclassified)
KPS3271-contig_6	37,003	51.8%	*Klebsiella* phage Kp4856-1 (Unclassified)
KPS3995-contig_8	42,804	51.6%	*Klebsiella* phage ST974-OXA48phi18.2 (Unclassified)
KPS4431-contig_51	25,748	53.4%	*Escherichia* phage ev017 (*Jouyvirus*)
KPS4983-contig_15	38,044	53.5%	*Klebsiella* phage ST846-OXA48phi9.1 (Unclassified)
KPS6186-contig_1	22,351	51.8%	*Klebsiella* phage P12620-1 (Unclassified)
KPS6191-contig_6	38,650	52.7%	*Escherichia* phage PDP110_P2 (*Hendrixvirinae; Wanchaivirus*)
KPS78133-contig_3	42,102	50.7%	*Stenotrophomonas* phage vB_SmeS_BUCT709 (Unclassified)
KPS7939-contig_9	45,850	52.9%	*Klebsiella* phage ST512-KPC3phi13.1 (Unclassified)
KPS8155-contig_2	49,994	52.4%	*Klebsiella* phage 2b_LV-2017 (Unclassified)
KPS8329-contig_8	40,215	52.0%	*Klebsiella* phage RCIP0114 (Unclassified)
KPS8961_contig_2-2	40,432	52.3%	*Klebsiella* phage vB_Kpn_Chronis (Unclassified)
KPS9086-contig_11	33,216	55.5%	*Klebsiella* phage ST101-KPC2phi6.2 (*Peduoviridae; Reginaelenavirus*)

## Data Availability

Data are contained within the article and the Supplementary Materials.

## Supplementary Materials

The following supporting information can be downloaded at: https://www.mdpi.com/article/10.3390/v17050623/s1, Figure S1: Maximum-likelihood phylogenetic tree based on amino acid sequences of TLS. Bootstrap values are shown near the nodes. The scale bar indicates 0.5 estimated substitutions per site, and the tree is midpoint-rooted. *A. baumannii* KL type is indicated in the legend. Prophage regions are named according to the contig of the genome assembly in which they were identified; Figure S2: Maximum-likelihood phylogenetic tree based on amino acid sequences of TLS. Bootstrap values are shown near the nodes. The scale bar indicates 0.5 estimated substitutions per site, and the tree is midpoint-rooted. Representative prophage regions are colored gray; Figure S3: AlphaFold models of putative BHPs in prophage regions KPS4983-contig_15 and KPS09509-contig_15. Rainbow coloring uses a gradient where the N-terminal end is blue and the C-terminus is red; File S1. Representative prophage regions from *A. baumannii*; File S2: Representative prophage regions from *K. pneumoniae*; Table S1: Completeness and metrics of *A. baumannii* and *K. pneumoniae* genome assemblies assessed using CheckM2; Table S2: Antibiotic resistance/susceptibility profiles of *A. baumannii* and *K. pneumoniae* isolates.

## Abbreviations

The following abbreviations are used in this manuscript:

bp	base pair
MCP	Major capsid protein
MDR	Multidrug-resistant
PDR	Pandrug resistant
PI	Pairwise identity
PTDE	Phage tail depolymerase and esterase
RBP	Receptor-binding protein
TLS	Terminase large subunit
TFP	Tail fiber protein
TSP	Tailspike protein
XDR	Extensively drug-resistant
ZOT	Zonular occludens toxin
