# Supplementary figures and images for: Acinetobacter baumannii and Klebsiella pneumoniae Isolates Obtained from Intensive Care Unit Patients in 2024: General Characterization, Prophages, Depolymerases and Esterases of Phage Origin
Source: Viruses. 2025 Apr 26;17(5):623. doi: 10.3390/v17050623 (PMC12115436; doi:10.3390/v17050623)

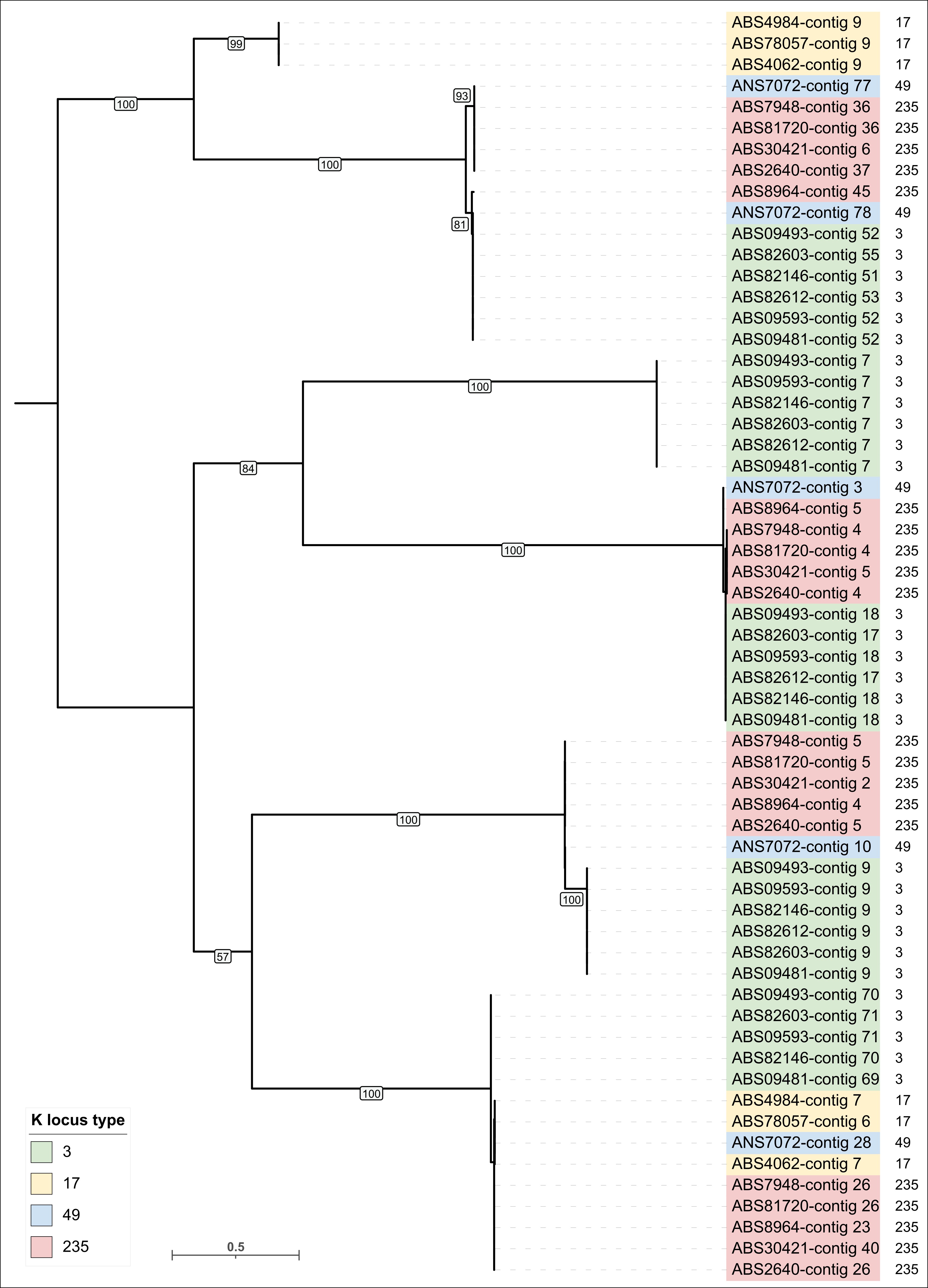

Supplement: Supplementary file 1 [file viruses-17-00623-s001.zip › Figure_S1.jpg]

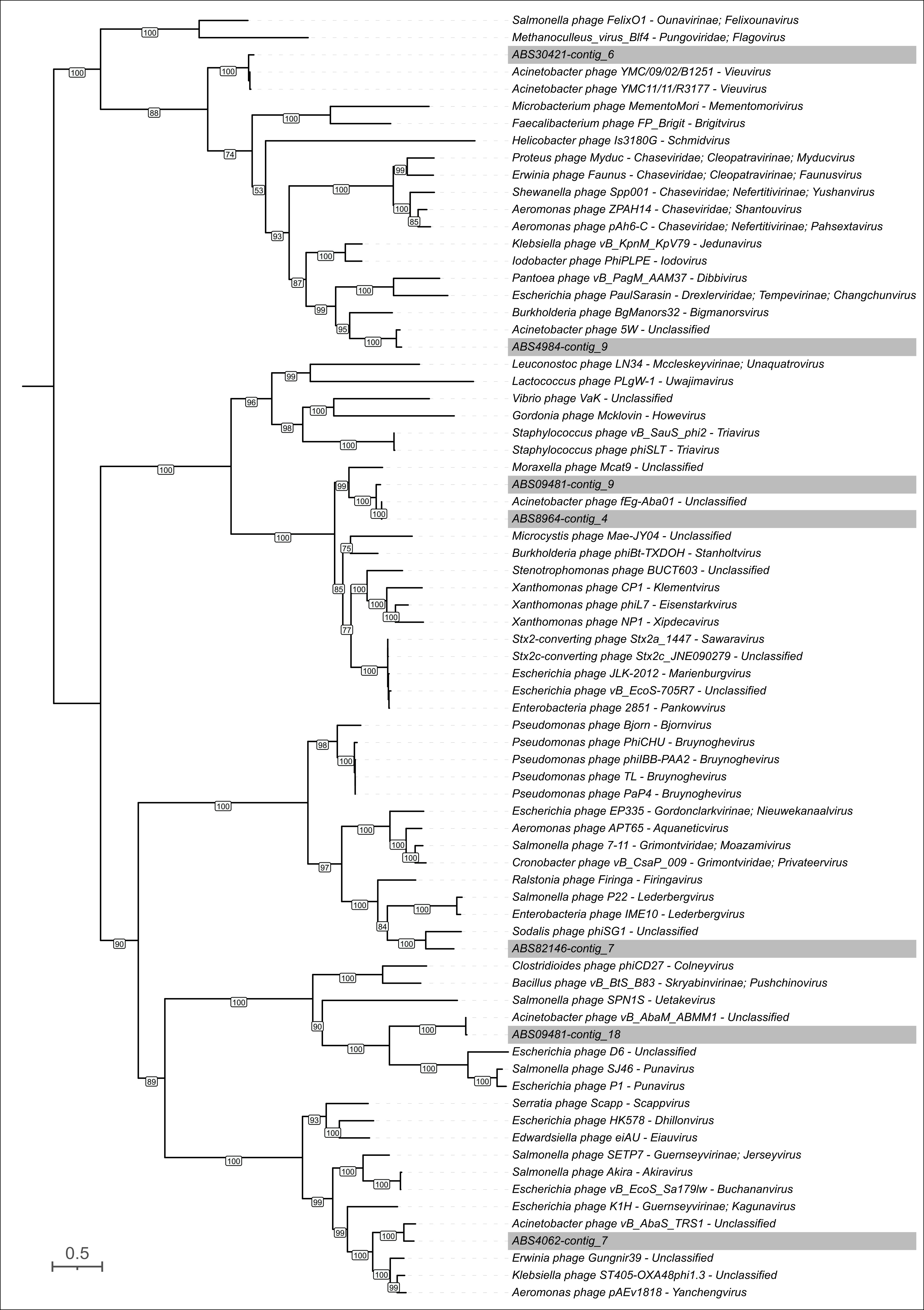

Supplement: Supplementary file 1 [file viruses-17-00623-s001.zip › Figure_S2.jpg]

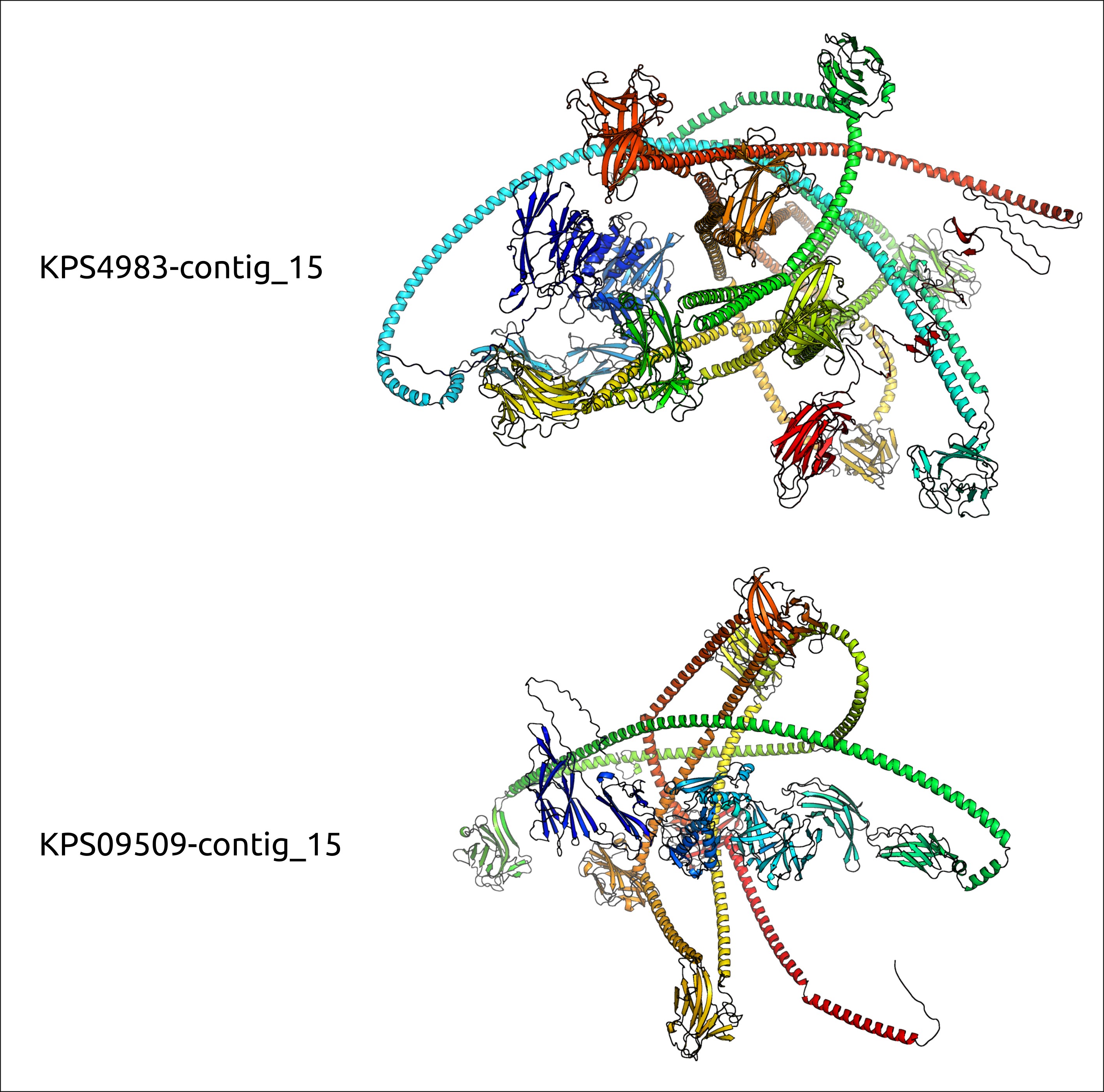

Supplement: Supplementary file 1 [file viruses-17-00623-s001.zip › Figure_S3.jpg]
